# Grand challenges in visceral surgery

**DOI:** 10.3389/fsurg.2022.1005046

**Published:** 2022-08-25

**Authors:** Gabriel Sandblom

**Affiliations:** Department of Clinical Science and Education Södersjukhuset, Karolinska Institutet (KI), Solna, Sweden

**Keywords:** liver surgery, artificial intelligence, global surgery, regenerative medicine, telemedicine

“The good physician treats the disease, the great physician treats the patient who has the disease.” (William Osler). Removal of the organ affected by a neoplastic, infectious, or inflammatory process has been the primary aim of visceral surgery for more than a century. Recent developments, however, have enabled visceral surgery to aim not only at an “ectomy”, but also to restore function of the organ. Effective collaboration with anaesthetists, radiologists, oncologists, gastroenterologists, and other specialists involved in the care of patients with a disorder of the gastrointestinal tract has made it possible to carry out visceral surgery taking all aspects of tumour biology, physiology, and gastrointestinal function into account. Bariatric surgery leads not only to weight-loss, but also to prolongation of life as well as curing diabetes and other conditions associated with obesity, commonly named the metabolic syndrome. Improved understanding of the regenerative capacity of the liver has made it possible to extend the boundaries of hepatic surgery with little risk for liver dysfunction. Endoluminal surgery has made it possible to carry out advanced interventions without the need for resecting segments of the gastrointestinal canal (1). Enhanced recovery has greatly reduced the physiological impact of surgery on the patient (2). Modern hernia surgery focuses not only on closing the hernial defect but also maintaining abdominal wall function.

Nevertheless, visceral surgery is hazardous and feared by the general public who associate it with blood, pain, and a long stay in hospital. The ability to surgically cure malignant as well benign conditions today, that were considered way beyond any cure a few of decades ago, is not widely understood by the average man on the street, and there is a great need to communicate this progress to the community at large.

Regenerative medicine is already widely practised in plastic surgery and will probably become a part of visceral surgery in the foreseeable future. Tissue engineering has enabled repair or regeneration of damaged or defect tissues. The development of bioengineered synthetic degradable scaffold materials may enable us to at least partly replace visceral tissues (3). Improving the functional capacity of regenerating tissue is still a great challenge, but with an increasing understanding of tissue architecture, this may be accomplished in the future.

Telemedicine and telesurgery have overcome the problem of physical distance between the patient and the surgeon who can perform the specific procedure required. Advances in robot surgery, fibreoptics, and computer technology will probably surmount the geographical obstacles that limit patient access to best possible care. This could well turn out to be an effective way of centralising complex surgery, thereby improving patient safety. However, interactions between patient and surgeon should also satisfy the basic needs of healthcare *i.e.*, support and comfort, which is difficult to achieve without face-to-face contact. Future surgical care should thus focus on a trade-off between advanced technology and traditional establishment of confidence between surgeon and patient.

Global surgery is also undergoing rapid changes. Many parts of the developing world, with their enormous populations, have traditionally lacked access to even basic surgical care, in particular South America and East Asia. Many such countries now have the economic resources to finance the basic surgical care of millions of people. Even though the technology presented in cutting-edge reports mainly comes from high-income areas, lessons learnt from the development of minimally invasive techniques regarding anatomy, physiology, and biomaterials may also be applied in healthcare systems lacking access to advanced technical equipment.

Future development of surgery on the global scale should thus focus on sharing experience and knowledge between high- and low-income areas. In many poor countries, access to necessary resources is not the primary limitation but rather the lack of surgeons. The traditional way of providing basic surgical care in such countries has been to export volunteers and donate resources. This principle is gradually being replaced by local organisation of basic healthcare and education of local expertise. To set standards of care at a reasonable level, needs and limitations must be defined by the local population.

Ideally, research in visceral surgery should consider the global inequity in access to resources, equipment and education (4). Global access to high-quality surgical care is unequal. Nevertheless, we all have a common goal to update our knowledge and understanding of anatomy, physiology, and methods to restore function. Mesh material used for hernia surgery in the Western world has been developed to meet all demands regarding the reduction of mesh-related complications. The high cost of these implants prohibits large scale import in areas where resources are limited. However, research has established basic repair techniques such as the optimal anatomical position to apply mesh. This knowledge may be extrapolated to poor areas where cheaper meshes are produced locally (5). Greater understanding of the anatomy of the bile ducts and gallbladder will improve the safety of gallstone surgery, regardless of where it is carried out. Similarly, our understanding of best timing and surgical approach to achieve radical resection of malignant visceral tumours applies anywhere in the world, even when advanced equipment and oncological resources are lacking (6). The impact of the stress response on circulation, immune system, neuroendocrine system, homeostasis, and the tumour itself is universal, regardless of local economic circumstances. International research collaboration should lead to new treatment strategies with improved patient safety on a global level regardless of differences in access to resources and qualified surgeons.

There is a widespread conviction among surgeons that visceral surgery, a speciality requiring craftsmanship, will continue to be unaffected by the recent hype in artificial intelligence. Artificial intelligence, however, is here to stay, and it is crucial that the future development of AI in surgery is directed by the needs of surgeons and patient groups. Studies have shown that machine learning may improve clinical decision-making in complex cases with multiple comorbidities (7, 8). In any domain where pattern recognition is required, machine learning algorithms may help provide more accurate predictions of treatment outcome. The massive accumulation of surgical data in numerous registers together with machine learning will probably play an ever-increasing role in the organisation of healthcare for patients in need of surgery. Artificial neural networks have been used to predict treatment outcome and to improve risk stratification. Other fields where artificial intelligence is gradually increasing include the use of robots to carry out well-defined isolated tasks such as suturing intestinal anastomoses, and navigation in liver surgery using augmented reality.

The future of visceral surgery is difficult to predict. Great changes are expected in the complex surgical management of malignant conditions as well as minor routine surgery. Technical advances available to only a small rich minority will hopefully spread globally as surgical equipment is produced on a larger scale with consequent fall in prices. Future surgical research should avoid disruptive innovations i.e., new techniques fueled by the attraction of fancy technology rather than unmet patient needs. Ideally, the surgical community should set the goals of future developments, only relying on financial support from the industry when necessary.

## References

[1] Peltrini R Imperatore N Di Nuzzo MM Pellino G. Towards personalized treatment of T2N0 rectal cancer: a systematic review of long-term oncological outcomes of neoadjuvant therapy followed by local excision. J Gastroenterol Hepatol. (2022) 37(8):1426–33. 10.1111/jgh.15898 35614027 PMC9545053

[2] He L Lu L Su S Lin Q Sheng C. Top 100 most-cited articles on enhanced recovery after surgery: a bibliometric analysis and visualized study. Front Surg. (2022) 9:845946. 10.3389/fsurg.2022.845946 35599804 PMC9114349

[3] Adil A Xu M Haykal S. Recellularization of bioengineered scaffolds for vascular composite allotransplantation. Front Surg. (2022) 9:843677. 10.3389/fsurg.2022.843677 35693318 PMC9174637

[4] Siddiqui NA Raees MA Khan RN Zafar F. Barriers in surgical research: a perspective from the developing world. J Pak Med Assoc. (2022) 72(Suppl 1):S97–S102. 35202379 10.47391/JPMA.AKU-19

[5] O’Brien J Sinha S Turner R. Inguinal hernia repair: a global perspective. ANZ J Surg. (2021) 91(11):2288–95. 10.1111/ans.17174 34553473

[6] Theyra-Enias H Tumba N Popoola OB. Management and outcome of colorectal cancer in a resource-limited setting: Ahmadu Bello university teaching hospital, Zaria, Nigeria. Niger J Clin Pract. (2022) 25(6):923–30. 10.4103/njcp.njcp_1948_21 35708435

[7] Zhao PY Han K Yao RQ Ren C Du XH. Application status and prospects of artificial intelligence in peptic ulcers. Front Surg. (2022) 9:894775. 10.3389/fsurg.2022.894775 35784921 PMC9244632

[8] Taha A Enodien B Frey DM Taha-Mehlitz S. The development of artificial intelligence in hernia surgery: a scoping review. Front Surg. (2022) 9:908014. 10.3389/fsurg.2022.908014 35693313 PMC9178189

